# Autonomous Robots for Services—State of the Art, Challenges, and Research Areas

**DOI:** 10.3390/s23104962

**Published:** 2023-05-22

**Authors:** Marius Misaros, Ovidiu-Petru Stan, Ionut-Catalin Donca, Liviu-Cristian Miclea

**Affiliations:** Department of Automation, Faculty of Automation and Computer Science, Technical University of Cluj-Napoca, 400114 Cluj-Napoca, Romania; marius.misaros@aut.utcluj.ro (M.M.); ovidiu.stan@aut.utcluj.ro (O.-P.S.); ionut.donca@aut.utcluj.ro (I.-C.D.)

**Keywords:** autonomous, robotics, assistance, connected, simulation, adaptive systems, privacy, smart mobility, intelligent systems, mobility services, proximity mobility

## Abstract

It has been almost half a century since the first interest in autonomous robots was shown, and research is still continuing to improve their ability to make perfectly conscious decisions from a user safety point of view. These autonomous robots are now at a fairly advanced level, which means that their adoption rate in social environments is also increasing. This article reviews the current state of development of this technology and highlights the evolution of interest in it. We analyze and discuss specific areas of its use, for example, its functionality and current level of development. Finally, challenges related to the current level of research and new methods that are still being developed for the wider adoption of these autonomous robots are highlighted.

## 1. Introduction

Autonomous robots have become an essential component in some areas of personal assistance and manufacturing, which means their numbers are constantly increasing as they are being used in more and more areas of activity. However, the next major challenge for robots is to improve their applications and communication with the external environment for fully autonomous control [1].

Human–robot interaction is a multidisciplinary field that aims to “analyze, design, model, implement, and evaluate robots for human use”. Although there has been a great deal of development in studying how humans interact with robots in the course of an activity, this part of the technology has started to gain more momentum in recent decades, a process that has led to the increasing use and growing impact of these robots in society, which has also led to an increase in the popularity of personal assistant robots (robots that help people by facilitating the performance of personal activities) and in the manufacturing sphere (robots used in production units). This period of development is justified by several characteristics that define the concept of faster development. The first characteristic is the fact that studies focusing on a longer period of time produce results as broad and structurally sound as possible. Another key feature relates to the robust technology we currently benefit from, which allows a high degree of self-control, which facilitates the interaction of personal robot users with their robots over a longer period of time [2].

In [3], several characteristics are highlighted with respect to the classification of autonomous robots. The first feature is determined by the concept of “performing actions automatically”, which is a necessary element in the way a robot works, but which is also visible and required in other simpler systems called automata. The functionality of the mechanism is also obvious in other systems, such as dishwashers and autopilot planes, but to perform these tasks, there are machines that make fine and easily interpreted distinctions between what is meant by an autonomous system and what is meant by classical robots.

The necessary building components of a robot without which it could not perform its operational mission are sensors. A large proportion of automated systems lack these elements, which tells us that these robots lack the ability to adapt their action to the environment in which they operate.

Autonomous robots are present in environments in which human control is impossible or is not cost effective. They autonomously perform physical and computational behaviors depending on the task they are required to perform. The adoption of this technology has a very wide reach at the moment, integrating a multitude of fields such as mechanics, artificial intelligence, biology, and natural systems [4].

The evolution of the ecosystem is pushing for increasing the adoption of autonomous robots, and they are being integrated into most fields with the aim of bringing more efficiency into interactions of all kinds [5]. The way humans and robots cooperate is becoming more and more personalized with the ability to provide assistance in different areas. They are designed with the aim of reacting autonomously and safely according to the task. One aspect that needs to be taken into account for these types of systems is that they need to be rational and have a well-developed learning capacity [6].

In [7], developments in the field of autonomous robots that generally adopt evolutionary learning are presented, referring to learning through novel experiences, which then automatically become part of the robot’s ability to perform similar tasks to those already performed. Understanding this approach, the methods used are not specialized for the purpose of solving a task, but rather the development of efficient algorithms to in-crease learning specialized for a broader goal. In this area, there has been a series of research papers on how robots learn skills [8,9].

**Figure 1:**
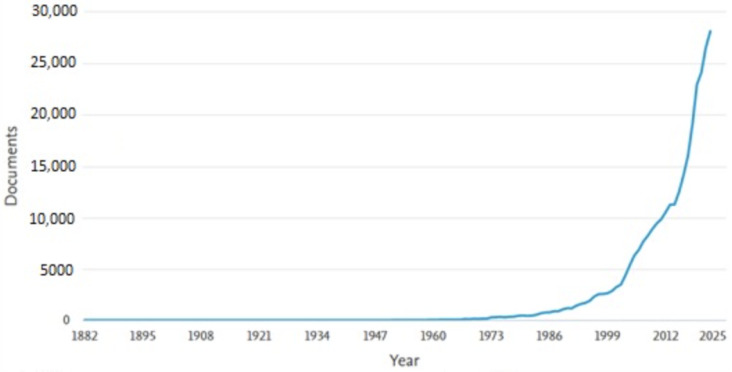
The trend in the volume of scientific literature pertaining to autonomous systems over time.

Following an analysis of the database provided by Scopus, Figure 1 shows an analysis of the level of interest in this field from 1982 to the end of 2022. From 1986, interest in this field started to increase gradually until 1999, followed by a period of explosive growth with a high level of interest until the end of the analysis.

**Figure 2:**
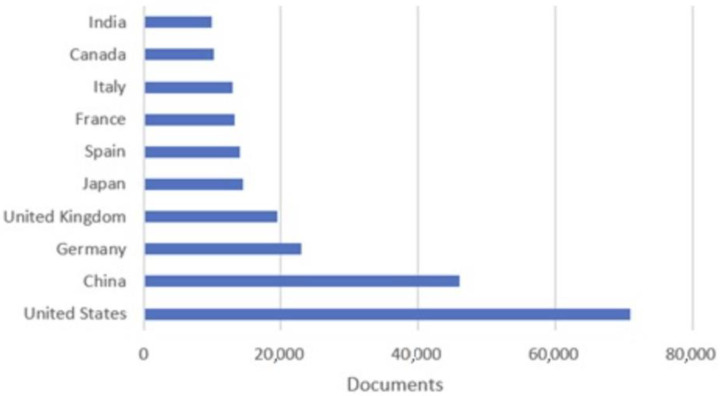
Country classification of the level of scientific interest in the field of autonomous systems.

A study based on the interest in this field can be seen in Figure 2, in which a ranking of the top 10 territories with the highest number of publications in this field between 1882 and 2022 is presented.

## 2. Assistive Robots

In the past few years, assistive robot services in unsafe environments have seen increased adoption rates. These types of robots have been adopted and used in areas such as shops, hospitals, museums, and even office buildings. For robots to be integrated into environments for the purpose of assistance, they need to have efficient navigation and safety in terms of their area of activity [10].

The notion of an assistive robot refers to the idea of cooperation by providing support in various actions of human users [11]. Research has been carried out in the fields of rehabilitation [12,13,14,15], companionship [16,17], mobility [18,19,20], and assisting a physically disabled user [21,22,23].

A very important aspect of entirely autonomous technology is that it would have the ability to fulfill the potential of social interaction in a fully autonomous way, able to relate to multiple users of a personal robot. These types of interactions have a fairly high degree of difficulty; therefore, it must exhibit the ability to manage and identify the needs of a group of users throughout such an interaction. However, in order for such a robot to be effective in implementing and carrying out the various activities that are characteristic of a particular group of people, it must be competent in independently managing and planning daily activities, taking into account the schedule that these users have. In contrast to these aspects, which are difficult to track and fulfill, a number of social robots have recently been developed to interact with multiple users, with certain responsibilities such as guiding visitors around a museum or even replacing a barman.

In order to carry out a visit to a museum, these robots used a tour based on a planning algorithm to identify which topics are approachable according to the knowledge it possesses, while taking into account the interest of the group and also the topics disseminated by the other robots up to the time of the meeting. Additionally, they have the capacity to switch individuals across existing groups if particular individuals may profit from greater information in another group that is run by other robots.. This experiment involved interactions between two robots and two virtual groups of people [24].

**Figure 3:**
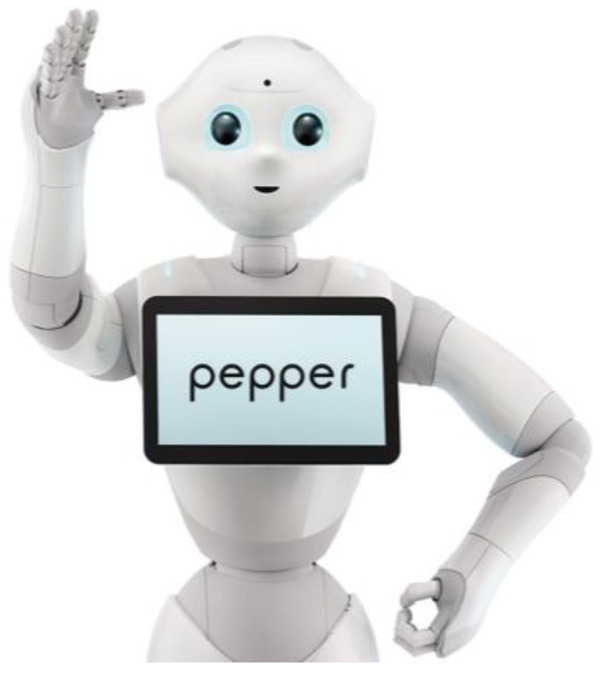
Pepper robot.

In [25], an interaction mode was studied by conducting a simulation with a Pepper robot (Figure 3) which had the ability to detect groups of people and their orientation, then identify a position in which it could integrate those groups.

Assistive robots come with a number of advantages and disadvantages, the main advantage being that they significantly reduce worker fatigue, and a disadvantage of these robots is that the interaction time between workers is significantly reduced. Most of the research in this area has focused on human–robot interaction [26,27,28,29].

In [30], it is pointed out that, so far, most applications have only one person responsible for their functionality in the operating loop, which is not very favorable when it comes to long-term or remote operations. With embedded intelligence, the aim is to increase autonomy so that social robots are independent in planning the actions they need to take to achieve their described goals. They need to be able to recover from malfunctions that occur or that happen under uncertain conditions but also have the ability to adapt easily to changes that may be encountered. Therefore, the study of its trajectory and the dynamic planning of achievable tasks are rather important aspects in the development of autonomous robotics. The scope of development of robots is expanding more and more nowadays, accelerating toward human–robot interaction. These types of existing applications can be found in areas such as home care and even in entertainment interactions [31].

In [32], it is highlighted that these robots have the ability to detect their own environment and also the state of operation. These capabilities are learned from data acquisition based on the functionality and integration of sensors in systems, such as a camera that allows a robot, based on the acquired information, to coordinate its next movements.

Current robot navigation is based on a particular trajectory of a binary map; most of the time, the robot scans the environment using simultaneous mapping and coordination, segmenting the workspace into two parts: fixed obstacles and free space. There are also some integrated algorithms that create a ternary map (composed of fixed spaces, moving obstacles, and fixed obstacles). Robots operating in a human-interacting environment need to have a better knowledge of their environment in order to have autonomous navigation in terms of avoiding temporary obstacles that may intervene in their reach [33].

In [34], Cosar exemplifies a method by which an assistive robot comes to the aid of elderly people to enable them to perform certain activities. This robot has the ability to identify users through a thermal camera and an RGB-D camera. By combining the two cameras, upper-body detection is achieved with 70% efficiency. One camera refers to the use of 2D laser devices, as in [35], which aimed to identify obstacles and the region of interest. In [36], Ruwanthika used this method for robot localization, and in [37], this approach was used for scanning people in unknown environments, in which the scans were focused first on face identification and later on the body. Ge [38] dealt with the subject of children with ASD (autism spectrum disorder) by proposing a robot in a therapy room that identifies children’s interests or disinterest during treatment by analyzing their movements as engagement or disengagement patterns. To improve the safety of elderly people, Dimitrov [39] conducted a study in which a body analysis signaled an unexpected fall of an individual, which was achieved by implementing an algorithm on the robot PARbot. In [40], Theissen uses RGB-D in order to make robust and efficient maps for certain large-scale indoor environments; for more applications, we can also find this approach in [41,42].

O’Kane and Boccanfuso [43] have come to the aid of children suffering from autism by designing a humanoid robot that interacts with these children with the aim of improving their attention and encouraging verbal and non-verbal communication. In [44], an application was developed with the aim of benefitting human–robot communication by tracking gestures and faces, facilitating non-verbal communication. Cilmi presents in [45] an example in which the robot has considerable tracking capabilities by changing the position of its neck.

In [46], by means of RGB-D cameras, an algorithm implemented on a Pepper robot aimed to identify daily activities in a person’s life, such as the following: talking on the phone, drinking water, rinsing one’s mouth with water, writing on a blackboard, brushing one’s teeth, opening a pill container, stirring, relaxing on the couch, working on a computer, and wearing contact lenses. Jean Massardi [47] implemented an algorithm called PARC on a personal assistance robot which aimed to help people with disabilities to complete daily activities successfully.

Samuel et al. combined an analysis, sharing, and constraint clocks to achieve the shared control of assistive robots [48]. From what we have gathered, it is highlighted that Constraint Action Templates (CATs) are the first to allow a symbolic representation of these action sequences that can be shared and used for control and autonomy. Additionally, in this paper, CATs have been used in order to validate the successful completion of tasks.

Several social robot researchers have predicted that in the future, this will become a large-scale field, and the future will be shaped by fully autonomous social assistive robots. At the same time, researchers have encountered a discrepancy between the automatic level of autonomy (LoA) and social assistive robots, which leads to the conclusion that LoA technology is not necessarily a very good technology for fully equipped social robots. Thus, several researchers have chosen teleportation as the main technology for assistive social robots. Even though this concept involves teleoperator work, this technology fits most areas in which SARs operate [49].

Social assistive robots (SARs) are used in many areas, including the treatment of anxiety in children. These robots have been shown to have a high potential to treat this problem. A year-long study was conducted on a group of children aged 10 to 12 to determine how effective these robots are. Based on these elaborate studies, it has been shown that social assistive robots have a high capacity to help children with anxiety, whether they possess therapeutic qualities or not [50].

Irean [51] discusses the topic of assistive robots in terms of the constraints of using such robots in a classroom setting. The level of use has been established through several experiments in which safety and cost are factors that can act as constraints on the adoption of these robots.

The constraints in the implementation of features which can be encountered when discussing the adoption of these assistive robots in dynamic spaces, in terms of navigation, the detection of new objects, and localization, present complexity in their development. Safety systems regarding the specifications of assistive robots must include sterilization so that they do not become an unintended contaminant, for example, by spreading disease in an epidemic. From the point of view of cloud technology and communication, a major risk is their low security which would allow access to the database and the extraction of patients’ personal information for malicious purposes. An important aspect regarding the technical part of the robot is also directly related to its physical appearance because the user’s expectations at the moment of interaction are directly proportional to its appearance. Most mobile assistive robots benefit from a wheeled motion system which leads to less mechanical complexity and control [52].

**Figure 4:**
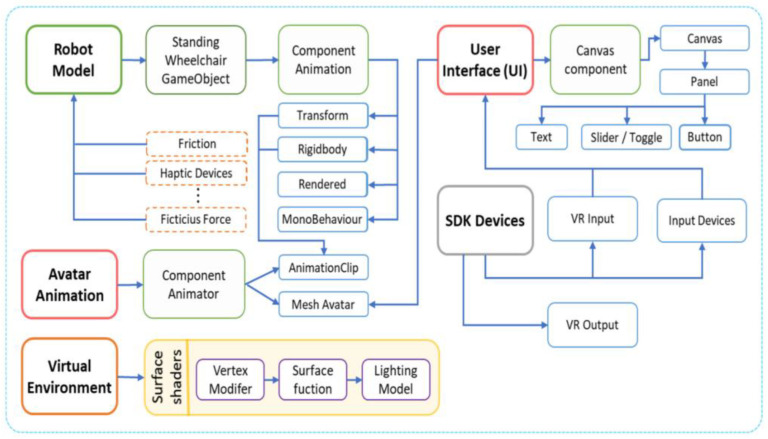
Scripting general scheme.

In [53], Jessica S. Ortiz studies the implementation of control algorithms for assistive robots that help people with motor disabilities or for rehabilitation. The practical implementation of this system was not possible due to the lack of a robotic system; therefore, a virtual reality system was used to simulate a standing robotic wheelchair to perform the tasks of the experiment. These virtual environments intended for rehabilitation must have the ability to allow human–robot interaction within any of the situations that may occur in real life, which favors its use in simulating other types of actions in terms of human–robot interaction. Figure 4 shows a scheme realized with external graphics executed on the Unity3D graphics engine.

Depending on the needs of the simulations, we have the ability to modify the system so that experiments can be performed within several scenarios for a set of as many data as possible in terms of the final result.

In order to interact with people with disabilities, such as deaf children, a humanoid robot has been developed that allows for the interpretation of signs made with one’s hands, arms, and head, specific to the language of the person with the disability. In this study, two of the most popular methods in sequential data processing were used: (1) based on the data provided by the Kinect sensor, an LSTM was applied in two layers; (2) and the second approach used a combination of an ANN with an HMM.

**Figure 5:**
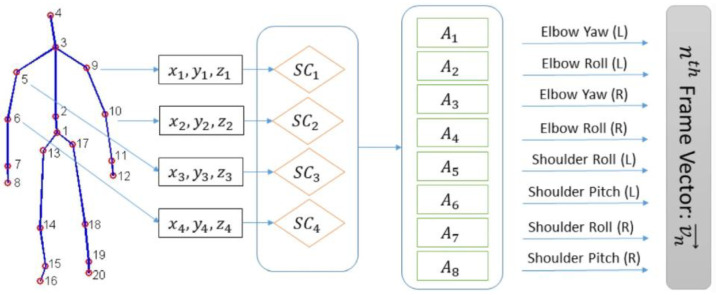
Preprocess of a Kinect data frame.

Data conversion and the generation of a motion file are highlighted in Figure 5, where four essential steps are covered, steps that can be used in making other types of applications:

1. The calculation of direction vectors using spatial coordinates (points 1–20 from Figure 5) in order to calculate the angles of the arcs;
2. The initial spatial coordinates of the arm ((x_1_, y_1_, z_1_) … (x_4_, y_4_, z_4_)) were used in order to extract and compute the rotation and roll angles of the elbow and shoulder (SC_1_, SC_2_, SC_3_, SC_4_), afterwards the basis vector is computed (A_1_, A_2_, A_3_, …, A_8_);
3. A certain vector is generated for each frame given by the Kinect sensor, a vector made from the following angles: left/right shoulder roll, left/right elbow roll, left/right shoulder roll, and left/right elbow roll;
4. The last step contains a movement file for each signature.

For the development of this application, the robot was physically and computationally limited, which led to the development of a low-cost real-time system [54].

Table 1 compares the publications used as our examples by addressing their functionalities, the tasks to be fulfilled, the working capacity, the user scope, and the camera performance of each autonomous robot included.

**Figure 6:**
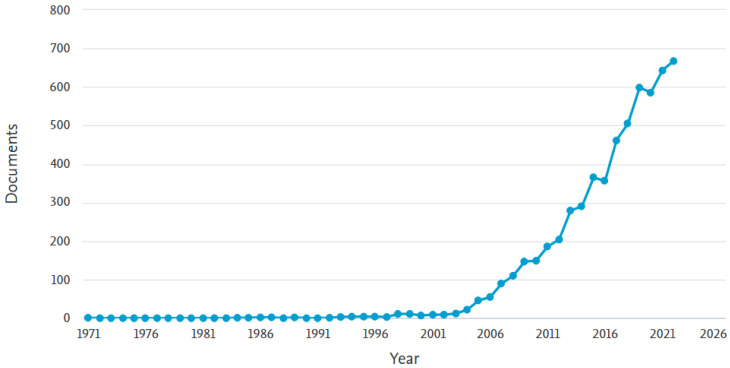
The trend in the volume of scientific literature pertaining to assistive robots over time.

An analysis between the years 1971 and 2022 is performed in Figure 6 to show the level of interest in autonomous assistive robots in the database provided by Scopus. Since 2003, the level of interest in this field has increased. This interest in assistive robots has continued to evolve gradually from year to year. By the end of 2022, according to the graph generated, it can be validated that research in this field has reached unprecedented heights, growing in the last decade by almost 100%.

**Figure 7:**
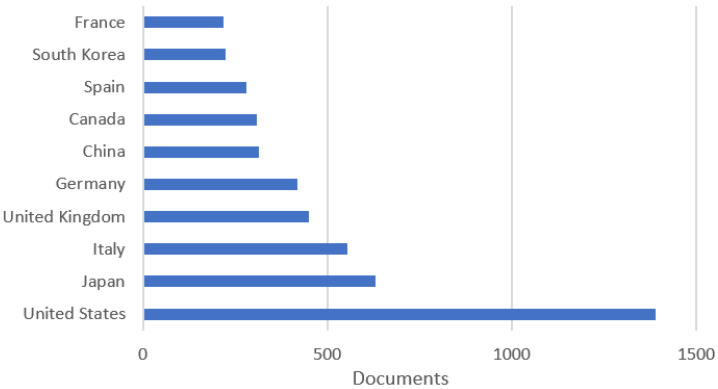
Country classification of the level of scientific interest in assistive robots.

Following an analysis of the Scopus database (Figure 7), a ranking of the top ten territories of greatest interest in this area is produced. Topping the ranking is the United States, which ranks well above the other territories. The countries in the next places gradually decline in interest, followed by those in sixth and seventh place, which have about an equal level of interest and are very close to that of the country in eighth place. The countries in ninth and tenth place also have an almost equal level of interest.

## 3. Autonomous Vehicles

Fully autonomous cars are now well past the stage of laboratory experimentation. This is due to fairly high levels of competition in the market and the desire of each manufacturer to make as autonomous a model as possible. Their models are becoming increasingly complex and well structured. Their automation consists of adding a new layer, i.e., the introduction of cognitive intelligence adapted to the platforms used for the vehicles [55].

The mechanical elements that make up a machine are currently seen as the building components of a machine, and now the software integrated into a machine is becoming the main development component. These software systems have a high degree of complexity determined by their very non-linear nature; within this system, the signaling of a one-bit error can bring down the whole system, but there are also cases in which an important error occurs that may not have any impact on the system [56].

In [57], standard vehicles are shown that have software systems implemented with certain rigid constraints in terms of safety, real-time processing, maintainability, and also failure rates. The degree to which software evolves to have strong autonomy requires rigorous management, which can lead to the avoidance of design flaws or misrepresentations of certain requirements in the final design. As smart cars become electrified and connected, vehicles will also start to emerge with very different looks and features, which may even lead to them not being considered as part of the car category.

In Table 2, some of the characteristics of autonomous cars are shown. All the features noted provide us with an overview of all their new capabilities, such as the following: being much cleaner, more energy efficient, safer, smarter, more pleasant, easier to drive, more innovative in terms of design, and last but not least, easier to travel with than the cars and trucks that have been developed so far [58].

**Figure 8:**
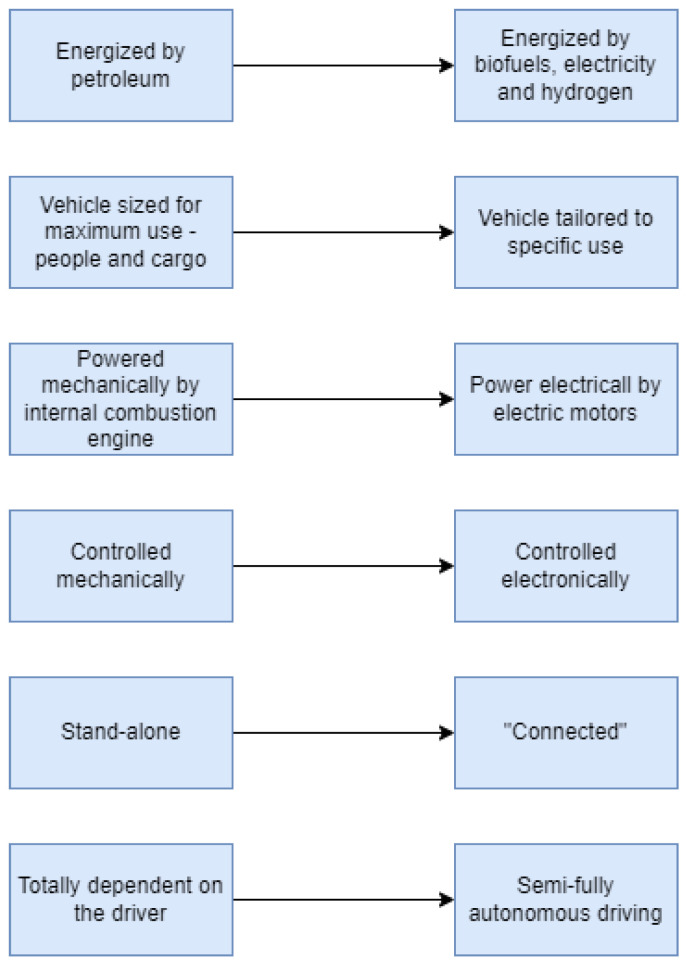
Transposition of characteristics.

The adoption of these new features of autonomous cars assumes a number of transpositions in terms of their evolution, as shown in Figure 8.

A major benefit that autonomous vehicles could bring is the ability to give people with disabilities the opportunity to travel on their own without the need for an attendant [59]. This will achieve the inclusion of these people into society and, at the same time, aim to significantly reduce the dependence on the control of manually operated vehicles. In order to realize this type of vehicle, it is necessary, first of all, to design and build a user interface in an inclusive way, so it is able to respond to a wide range of needs that different members of society may have. However, studies are still ongoing to develop such an interface, as the process is still in its infancy.

Autonomous vehicles are classified by the Society of Automotive Engineers (SAE) into six levels of autonomy that estimate their ability to perform driving tasks. These levels are from 0 to 5. At Level 0, the car has no automation and its entire control is the responsibility of the driver. At Level 1, the system provides assistance to the driver, such as acceleration or deceleration, by obtaining information from the environment. Level 2 contains partially autonomous systems, which, based on information received from the environment, provide steering and acceleration/deceleration control of the vehicle. Level 3 is defined as a conditioned automation that includes vehicles with the ability to execute throughout the system the aspects of the task of a dynamic connection with the condition that the driver responds in the case of an intervention. At Level 4, vehicles have a high degree of automation in which, throughout the system, the vehicle can have dynamic maneuverability without the need for driver intervention. Level 5 includes fully autonomous vehicles capable of adaptation and self-control in different environments comparable to the human drivers [60].

In order for a vehicle to be autonomous so that it can make decisions and recognize traffic signs, pedestrians, and other parties involved in traffic, it needs to learn. This perception is made on the basis of the data received from sensors [61,62]. Moreover, in [63], a convolutional neural network is illustrated; based on data extracted from a single camera, a vehicle learned to drive itself on local roads with or without markings and even on highways. This system implemented a type of automatic learning of processing steps, only having the steering angle of the human as its training. Another way of learning behavior is visible in [64], in which a learning-by-demonstration technique was used that develops polynomials based on state correspondences and state examples.

The problem of autonomous vehicles is approached from another perspective in [65], namely through trajectory-based traffic management (TTM) control and vehicle lane changes. The conducted experiment aimed to highlight the capability of TTM in changing different trajectories simultaneously for several vehicles. Another type of approach to changing the trajectories of a vehicle is treated in [66] by using images from SIFT features. This experiment used a stereo system added next to a car’s mirror to calculate the ego-motion system. In [67], the problem of traffic flow in heavy congestion was addressed by cancel the stop-and-go cars’ feature in case of traffic jams. This flow mitigation was addressed by controlling the speed of a given vehicle by comparing braking, acceleration, and fuel consumption respectively between experiments, which was shown to be possible by means of four mobile actuators.

In order to combine autonomous and human-controlled vehicles, Chen [68] explains the coalition principle, which refers to the distribution of data between vehicles for the purpose of receiving the next movements of traffic participants in front of the vehicle. He laid the foundation for future studies within the following four principles: collecting data provided by the five cars in front, calculating the capacity of a traffic network by the maximum average flow and critical density of vehicles, determining that a negative impact in the system will be a low number of high-speed vehicles, and determining a maximum average ratio for autonomous vehicles within certain density ranges. These types of robots have been adopted in various circumstances, such as in disaster management [69], missions in space [70], military missions [71], and as self-driving machines [72].

Hafiz et al. discusses safety, automated driving systems’ (ADS) regulations, and advanced driving assistance technologies (ADAS) for autonomous cars, such as vehicle-to-infrastructure communication, cooperative, adaptive speed control, LiDAR technology, RADAR, and others. Research has shown that CAV communications are of undeniable importance. The current state of the art shows that the evolution of these connected autonomous vehicles (CAVs) in terms of vehicle-to-vehicle (V2V) communication is very well developed with a wide range of vehicle-to-infrastructure (V2I) implications [73].

Autonomous vehicles have both strengths and weaknesses. Thus, a questionnaire has been carried out to determine what research needs to be done in this area for the world to benefit from fully autonomous vehicles in the future. A study found that some safety aspects, some safety and control algorithms, and route planning could be radically improved. It can be concluded that autonomous vehicles are already a way to the future and that Level 3 autonomous vehicles are already ready to be commercialized. This field is continuously developing, and further research and studies will lead to vehicles that go beyond those at the present level [74].

In order to move to autonomous vehicles as soon as possible, important aspects must be taken into account, namely, safety and control accuracy. As this area progressively develops, there has been a greater focus on ensuring comfort in an autonomous vehicle. A study was conducted based on a questionnaire containing questions on perception, navigation, positioning, and safety, i.e., the whole driving system. In conclusion, the future of autonomous vehicles is closely connected by the development of algorithms implemented on high-performance computers, similar to those of Tesla [75].

The population of cities is directly affected by the emergence of autonomous vehicles, by increasing or decreasing the number of inhabitants. The use of these types of vehicles brings added commuting benefits through the more efficient use of time spent commuting, leading to an increased number of people settling in non-urban locations. The effects of increased travel comfort and less time spent in traffic lead to more people relocating. This could lead to an 80–270% improvement in road capacity [76].

In [77], Felix presents a concept in which, after training and learning, a car is able to move autonomously in a non-urban environment. Route learning is achieved through lidar technology, with the car following a person moving in front of it to show it the desired route. On its return, the guide has the ability to correct the route while the vehicle navigates autonomously. This vehicle has a hybrid A* planner that generates routes at no cost, based on vegetation, local environmental features, slopes, and road probabilities. When it encounters an obstacle it cannot avoid, it uses Open Street Map data to identify a detour route.

Autonomous cars that benefit from a low-integrity system can ultimately benefit from a human driver’s experience of control. In the event of an ADAS software failure, there is the possibility that the driver may be able to overcome the error in order to drive the vehicle to a safe state [78].

One problem still encountered is the level of perception caused by unfavorable lighting conditions, e.g., large shadows can be mistaken for certain objects. At present, there are a lot of visual cues and thermal cameras that are integrated in order to enhance the performance of a system. However, in some situations, these systems may have difficulties in interpreting obstacles. Research suggests that no substantial amount of infrared camera tracking and detection algorithms are computationally efficient for real-time use [79].

A moment that confirms the increased attention to the perceptions of these sensors is the fatal accident created on 7 May 2016. The driver’s trust in the autopilot system was complete and he failed to intervene when it failed to intercept the white trailer of a white semi-truck due to white skies [80].

In [81], Azim highlights some of the current problems of autonomous vehicles, starting with the need to improve the algorithms responsible for the interaction between autonomous vehicles, human-controlled vehicles, and pedestrians, which are of major importance to update. A second aspect that needs to be improved refers to the adoption of an online decision-making process that brings a more equitable balance between model complexity and solution quality. Moreover, trajectory planning and decision making in dynamic places are absolutely necessary to improve. Some aspects such as noise or uncertainty can compromise planning. The way we plan as well as the distant horizon method must be developed by eliminating the risks involved.

The rapid development of technologies and their adoption in increasing numbers each year have accelerated the way autonomous vehicles are developed. Despite this, the existing limitations in cities are greatly hindering the promotion and application of these AVs. The following is a set of the characteristics that hinder the promotion of these types of vehicles:

- The absence of a high-level testing method and theory has limited their adoption;
- Cities have a dynamic environment which is inadequate to the participation of these vehicles in traffic;
- The infrastructure is not sufficiently developed to the extent that adoption is not a problem;
- The laws specific to autonomous driving need to be revised and clearly established according to new technologies [82].

At the moment, communication between vehicles is quite unreliable and limited with a large sequence of unprotected actions being used in the communication process. Another problem encountered is the miscommunication of the robot with the driver of the vehicle by misinterpreting traffic participants, and with increasing speed, the system becomes more and more insufficient in terms of perceived results [83]. Table 3 compares the possibility of success for one of the autonomous vehicles discussed and also highlights the presence (“🗸”) or the absence (“X”) of components used in each vehicle to achieve performance with a response time.

**Figure 9:**
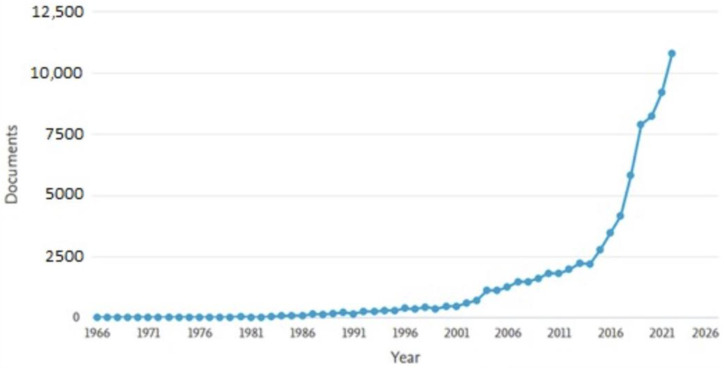
The trend in the volume of scientific literature pertaining to autonomous vehicles over time.

Next, an analysis between the years 1970 and 2022 on the Scopus database is shown in Figure 9. The first significant step in terms of the number of publications in the field of autonomous vehicles was made after the year 2003, and a constant level of interest was maintained until the year 2014. At the end of 2015, the level of interest spiked strongly and continued until 2022, increasing the number of published papers by up to five times.

**Figure 10:**
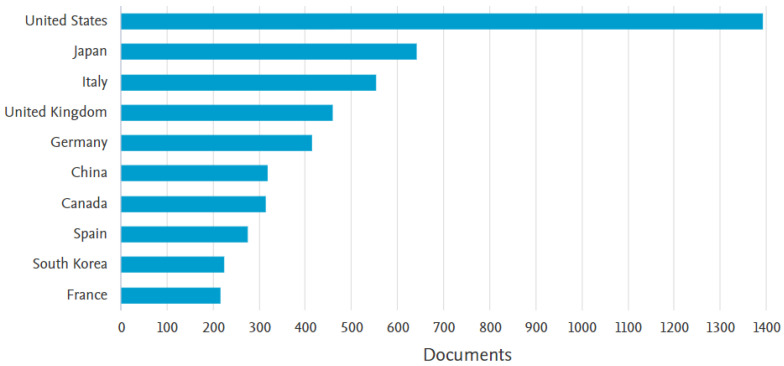
Country classification of the level of scientific interest in autonomous vehicles.

Figure 10 shows a ranking based on the top ten territories with the highest number of publications in the area of autonomous vehicles. The leader of this ranking is the United States, which has a substantial gap between the other countries in the ranking. Second place is occupied by Japan and third place by Italy, which had 87 fewer publications than Japan. The other seven territories in the ranking had graduatlly fewer publications, ending with France. The analysis was carried out on the database provided by Scopus.

## 4. Carry

**Figure 11:**
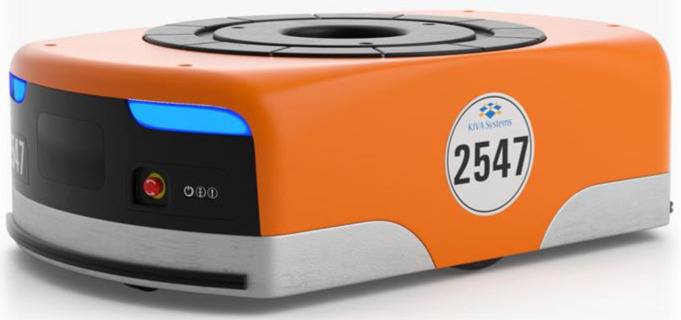
Carry robot.

It is well known that recently some major changes in energy systems have been established and noted, which have also had a significant impact on the field of conveyors. In light of this technology, in recent times many contributions have appeared towards the development of an algorithm with the main purpose of facilitating and optimally managing the control and monitoring of electric conveyors, dealing with an important topic of optimizing energy costs for certain hybrid systems. These optimizations have an increasing factor of importance, and one of the reasons is the dynamic energy tariff that leads to minimizing expenses. This aspect of expenses is a crucial one when it is discussed within a company. From the point of view of smart factories, autonomous systems responsible for performing certain predefined tasks in a factory with a high level of automation that also benefits from an intelligent communication infrastructure have the opportunity to have multivalent systems that coordinate the shaping of these systems [84]. Despite the fact that there are currently some very successful industrial conveyors, such as the Kiva System illustrated in Figure 11, many of the logistics and production processes within a factory are largely dependent on manually operated vehicles [85].

Currently, manually operated conveyors are the main cause of accidents in factories, which is why the automation of production is so desirable. These robots bring not only a substantial improvement in product quality but also an added benefit in terms of safety levels within factories. Furthermore, when it comes to scalability, situations in which bottlenecks occur are foreseen as additional problems; continuous improvement is needed to achieve more powerful and efficient algorithms [86]. The demand for placing as many conveyors as possible in factories is growing, so there are a number of challenges that may come into play for the large-scale adoption of these AVGs. Some of the most important parts are often implemented hastily and manually; e.g., route design is a crucial step that takes a good deal of time and has a high degree of error exposure when aiming to achieve objective perfection [87].

In [88], efforts to develop a prototype of an autonomous robot to transport tools and work, i.e., the raw materials within an enterprise to facilitate the work of employees, are described. This prototype for navigation is based on sensors and also on a camera for sensing the environment. Another model of robot, EcoBot-II, which is presented in [89], benefits from its own power supply by converting unrefined electrical energy into electricity.

The mode of communication and interaction between navigation robots was addressed in [90] by experimenting with two robots working cooperatively on cleaning. This experiment depicted building maps based on successful navigation and location communication. A study also carried out in the sphere of the interaction control of autonomous robot transporters [91] exemplified the efficiency of group transportation even though they have individual behaviors; however, in some situations, they suffer behavioral changes when interacting. The most favorable group behaviors allowed the robots to organize themselves into self-assembled structures, which generated evidence that self-assembly has the ability to provide adaptive value to individuals competing in an artificial evolution based on task performance. In [92], a method of collective transportation for multiple robots designated to operate tanks lacking advanced sensor and communication capabilities was studied. To capture information from the external environment, they relied on derivatives of the dynamics inherent in the interaction of robots with a common body, each intercepting information via a force sensor strategically mounted between the transported object and the manipulator. To address the topic of the joint manipulation of multiple robots, in [93], a system was exemplified that takes advantage of the opportunity to manipulate large and flexible objects without a special gripper. Since the robots were autonomous, coordination and association were achieved by communication and sensing, benefiting during transport from autonomous navigation.

In [94], a transport device was realized with the function of loading luggage by lifting and lowering it; furthermore, an analysis was performed to identify the position of the robot and track the trajectory to the desired destination. This robot was also equipped with a number of sensors that were designed to perform several activities during transport, such as identifying the parcel. According to [95], most robots so far have focused on robot positioning and product handling, neglecting their entry into dangerous areas, such as stairwells. More specifically, increasing safety is done by adding new sensors both in the external environment and on the robot, adopting a system called VLC (visible light communication) that uses the lighting in the building. The robot designed was called HOSPI and aimed to transport various instruments in a hospital setting. A paper that dealt with stair descent was [96], in which a robot was equipped with tracks, inertial sensors, and a monocular camera. For the treatment of this experiment, the following were considered: the optical flow, energy, and scene geometry. A real-time implementation of this study was on the iRobot Packbot robot, with the results of a real-world experiment being reported.

In [97], Masaki deals with the problem of transporting luggage/documents in hospitals where the construction of the MKR (Muratec Keio Robot, Murata Machinery Ltd., Fujisawa, Japan), which includes a collision avoidance technique, is exemplified. The method is based on potential future fields; several modules with different prediction times are estimated in parallel in order to modify the robot response according to its position, speed, and direction. The experiments concluded that the robot is able to move without problems in a real environment.

In [98], an autonomous system adapted to traditional AVGs is presented with the aim of operating in a safe way and reliably in crowded places. Usually, the reliability of AVGs is supported by some systems present in the infrastructure to support navigation, but not all environments benefit from such an infrastructure, which makes these systems not as accurate. This work aims to give AVGs the autonomy to handle cargo safely regardless of the environment and the load carried.

In industry, autonomous mobile robots (AMRs) are needed to streamline certain production processes. These types of robots rely on a combination of several types of sensors, powerful processors, complex locomotion systems, etc. AMRs may require a higher amount of energy to operate, but this pays off as they can be used 24/7 for continuous industrial processes [99].

In the context of the pandemic, which caused problems in all areas, the health service sector was one of the most affected. In order to be able to come to the rescue in the event of a similar situation, transport robots can be used to limit the spread of the virus. A study was conducted based on the implementation of transporter robots that have the ability to work on the front line with patients diagnosed with COVID-19. Their functionality was primarily based on the concept of continuous learning. Currently, the implementation of this learning strategy on medical transport robots has resulted in an approximately 2% reduction in deaths due to exposure to contagious viruses. In the future, based on the development of research, it is hoped to increase this percentage further by applying more complex methods of robot learning [100].

The adoption of these types of autonomous machines comes with a set of advantages that improve the quality, speed, and efficiency of handling and load distribution [101]. Integrating these types of conveyors within a warehouse can reduce unnecessary expenses, improve scalability, and lead to the accurate prediction of breakdowns [102]. The evolution of these conveyors combined with well thought-out automation can lead to a better customer experience that significantly reduces impacts on delivery networks [103].

These systems also have certain disadvantages, such as high costs, which makes the purchase of these products not extremely affordable. From the point of view of processing information and carrying out the activity, in some cases, there is also the possibility of interrupting the approach, which is caused by the use of a different technology [102].

In [104], the advantages of a centralized approach to task management are presented, in which tasks are correlated in batches of orders leading to a significant reduction in time. A uniform workload is also distributed between robots for a more efficient approach to individual requests.

A multi-robot system was integrated in order to streamline the operation of FMCG (fast-moving consumer goods) warehouses. This experiment was based on BudgeBOT robots built on two wheels with differential drive, which were equipped with cameras responsible for receiving feedback and correcting possible errors during transport. The architecture of the LOGISWARM system is based on a teleoperated leader and a number of tracking robots that use an overhead camera to detect their position and orientation. For the correct calculation of the position of each robot, the data are transmitted from the base station for error correction. In order to increase the working area, it is necessary to integrate several cameras for robot tracking and steering [105].

In [106], Yifan dealt with the topic of real-time task allocation in a smart warehouse system; a solution of the covariance matrix adaptive evolutionary strategy (CMA-ES) algorithm and a group task strategy were presented. The first step was to store random tasks so that their systematization and allocation was dynamic, having the opportunity to branch a large task so that the task division was fair and solvable in a more efficient way. It also addressed the adaptation of a control strategy specific to the cooperation mode in order to make a trade-off between energy consumption, workload, and waiting time. In the second step, when the task group was full, the optimization of the robot task allocation was applied in which the algorithm of the covariance matrix adaptation evolutionary strategy (CMA-ES) was used.

Table 4 compares the autonomous transporters in terms of the environment in which they operate as well as the speed and range they can reach. It is highlighted whether these transporters benefit (“🗸”) or not (“X”) from a camera in interpreting the environment.

**Figure 12:**
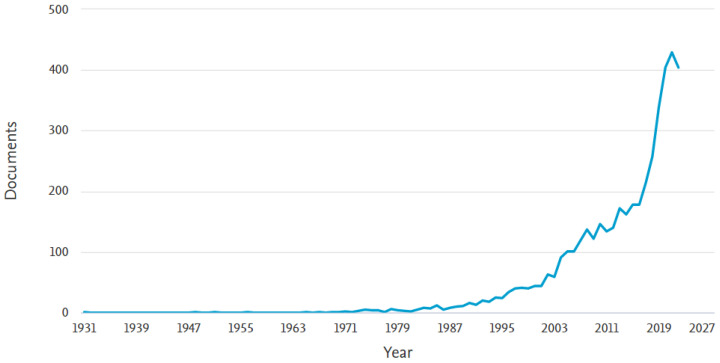
The trend in the volume of scientific literature pertaining to autonomous carriers over time.

A phased analysis from 1931 to the end of 2022 shows in Figure 12 the evolution of interest in this development area. The number of publications in this field changed significantly after the year 2000, when a major increase in the level of interest in scientific papers can be observed, which has been maintained until the end of the analysis; the number of publications multiplied up to 5 times. The analysis was carried out on the Scopus database.

**Figure 13:**
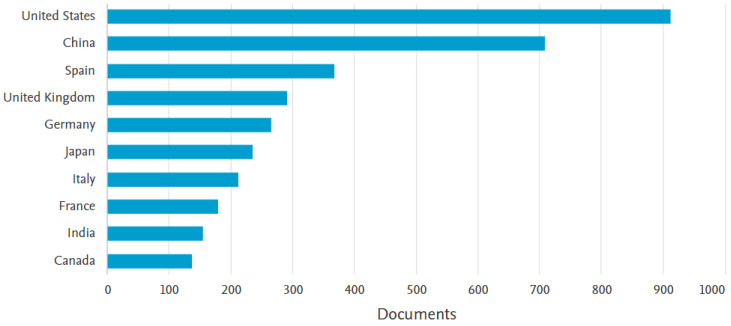
Country classification of the level of scientific interest in autonomous carriers.

In terms of the top 10 territories that have expressed an interest in publishing papers in this area, a ranking based on data from the Scopus library can be seen in Figure 13. The United States is the market leader in this field. China is in second place, followed by Spain, which has a significantly lower number of publications than China. The rest of the territories in the ranking have a uniform and gradual drop in interest.

## 5. Autonomous Manipulator

Throughout history, people have sacrificed themselves and taken on missions in uncomfortable and risky places to accomplish certain tasks, in some cases even paying with their lives. Accidents have been quite common, especially in developing countries. There are also currently a lot of activities that are still carried out by people who are at risk; for example, soap factories use chemicals that are harmful and lead to the sickness of workers who are exposed to them. The actions carried out by workers, despite being in an unfavorable environment, also have a repetitive cycle of work, which can lead to certain injuries or physical stresses on certain parts of the body. The need to eliminate these unpleasant situations for employees has led to the development of autonomous robots that can perform these tasks on their own.

The modernization and adoption of new autonomous technologies by manufacturers are intended to achieve one result in the first stage, namely the elimination of quality control because of human errors. Thanks to these systems implemented in the production process of a factory, a substantial improvement can be achieved, but more than that, they also have a strong impact on the reliability of the whole production system. Robots are specially designed so that they can withstand toxic operating environments as well as low or high temperatures and operate in explosion-prone places, which means that they protect the company from certain insurance claims, which are quite expensive [107].

In [108], researchers from the Politecnico di Torino developed an autonomous manipulator for assistance in hospital medicine. The robot was built with a customized omnidirectional platform, a robotic arm, sensors to monitor patients, and a tablet for interactions. To validate this robot, a series of tests were carried out in collaboration with the hematology department of Molinette Hospital.

**Figure 14:**
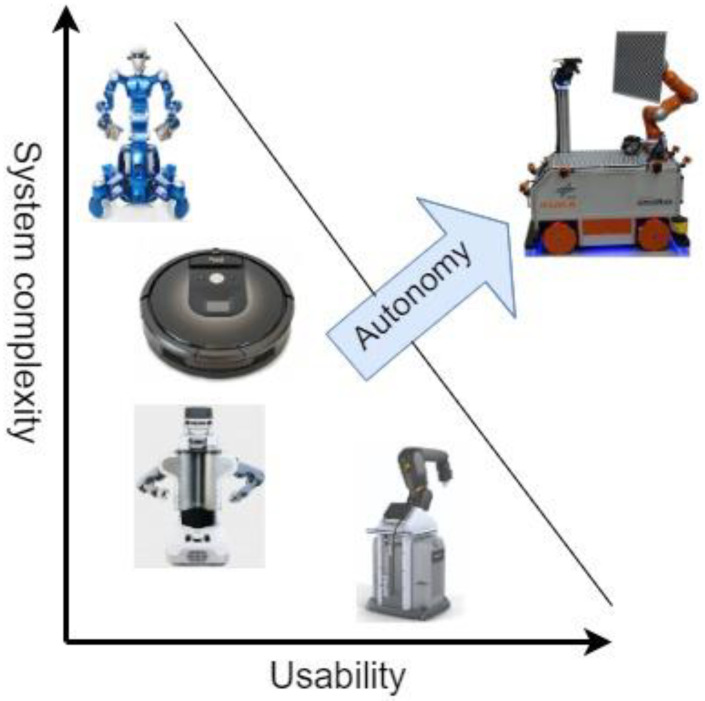
Comparison of classic robots with autonomous robots.

As shown in Figure 14, other complex systems can be used, e.g., DLJ Justin2 or PR21, which have the capability to solve complicated missions. At the same time, programming these systems requires a high degree of experience, which therefore leads to hiring a specialized person for this job, bringing an unfavorable cost to the company. There are also systems that can be controlled by inexperienced staff, such as BoschAPAS or iRobot Roomba, but these do not have the capacity to handle tasks such as transport and pick-up.

The fully autonomous manipulator has been a common topic in studies and research over many years. Nowadays, interest in this field is even higher; numerous vendors, such as Wil-low Garage PR2 (Willow Garage, Willow, NY, USA), Robotnik RB-1 (Robotnik Automation, Elche, Spain), PAL RoboticsTiago (PAL Robotics, Barcelona, Spain), KUKAomniRob III (KUKA AG, Augsburg, Germany), Fraunhofer Care-O-bot (Fraunhofer IPA, Stuttgart, Germany), and rob@work (Fraunhofer-Institut für Produktionstechnik und Automatisierung IPA, Stuttgart, Germany), have made systems available to both researchers and factories interested in this technology [109].

In [110], simplicity is exemplified in terms of building a handling robot that has the ability to be used in a general way. In the paper, an experimental way is trialed on the selection of waste bins by recognition, localization, and trapping. The main focus is on the identification of catch areas for object identification and location by addressing new design techniques, such as minimizing the number of catches.

In [111], the benefits of a micromanipulation system are presented in working with adhesion forces using an AFM (atomic force microscopy) probe. A theoretical analysis of rolling constraints was performed, resulting in the precise release of an object picked up by adhesion. Based on optical microscopy, vision control was performed, and based on a probe analysis, force control was performed. The problem of manipulating deformable objects was treated in [112], in which the segmentation of directions into haptic control primitives involving automatically vision-guided grasping and contact with the rigid medium was exemplified. Motion and force trajectories intervened at the moment of contact entry by acting as a reference.

Dov in [113] presented three objectives that must be met for autonomous manipulation: motion generation, object segmentation, and motion propulsion. Steps can be hindered when objects are not placed and are stored haphazardly in a pile. The demonstration in this publication was performed using a robot that aimed to select cluttered objects from a table and store them in specific compartments. A number of control strategies were developed in [114], with the aim of improving the performance of an artificial carrier when sliding an object from the gripper. The control methods within this component are independent, and combining them will increase the performance of the system. The transduction method uses the vibration that occurs when sliding and the touch sensor to detect the attempt of the object to fall from the hand. Although this approach is the basis of a prosthesis, this method can also be included in other activities carried out with robots.

In [115], an approach to autonomously manipulating tissues with anisotropic deformation is presented. This study has the main features of online estimation and learning, generating an independent implementation in terms of system calibration with respect to the robot and also the encountered deformation, a characteristic that is not favorable in the manipulation of an unknown deformable tissue by a continuous manipulator. Three different experiments were performed to observe the capabilities of the robot based on a da Vinci kit with a 5 mm instrument that had four degrees of freedom and a snake-like wrist, so that situations occurring in surgical schemes were executed, measuring flexibility, precision, and learning ability.

Approaches to autonomous handling have also been tested in underwater environments. In [116], an application was created that aimed to manipulate an arm at the bottom of the ocean; the objectives pursued were arms control, the visual tracking of objects, and safe grasping. According to [117], autonomous underwater manipulators are predominantly used in research activities; they are less cooperative in remote communication due to significant data transmission delays and benefit from a reduced bandwidth. This study aimed to improve the level of data transfer between control parties. In [118], a control for the manipulation of a GIRONA500 subaqueous dispositive based on kinematic control was presented. A task concurrency approach and a combination of redundancy according to task priority were used. The hierarchy of tasks to be completed and the strategies applied to achieve the proposed goal were also discussed.

In [119], an autonomous handling system was presented with the aim of helping people in a library by searching for, retrieving, and delivering a book from the shelves. Automatic recognition systems, grasping systems, and feedback of the force exerted in grasping were adopted to accomplish this task. Axelrod [120] addressed the problem of calibrating handling robots by addressing a method that applies the transformation reported to the location of detected objects in order to obtain a transformation of the commanded terminal effect without adding other external accessories.

In [121], Sandeep worked with linear manipulator systems that have the ability to be reprogrammed to adapt to product changes and are flexible to meet unique requirements. These types of systems have a much higher accuracy, making them suitable for simple and repetitive tasks. The prototype in this paper consisted of an n-link robotic arm that was mounted on a moving slide the length of a track. By using the Lyapunov control scheme (LbCS), new systems were developed that were based on acceleration in navigation in view of inaccessible target waves. In terms of linear manipulator limitations and singularities, these were artificially treated in the applied motion control scheme. This type of linear manipulator, due to its superior precision capability, can be adapted for assembly, packaging, or even in the intervention of the operator.

Manipulators have become staple components in adaptive assembly systems because they have increased flexibility and a high level of accuracy. A study was carried out based on a smoothing ant colony algorithm for the purpose of planning the trajectory of a gripper manipulator. A sampling curve B was specially designed to eliminate folding points and to traverse the desired directions without collisions; additionally, the algorithm used had an added benefit in terms of path planning efficiency and path quality. The experiments yielded the following: the shortest path was optimized by 22.1%, the number of paths without collisions increased by four times, and the execution time was reduced by up to 33% [122].

In [123], the tracking of mechanical systems was developed according to the required path to be followed by the manipulators with the aim of improving the accuracy of the system, which increases the safety factors when in use. A neural network was created to track the trajectory of these manipulators in order to improve the system for the synchronous adoption of several manipulators in performing several complex tasks. This study improved the co-ordination of several robotic arms and their correlation to achieve simultaneous robotic performance. Disturbances from the external environment and non-linear noise factors were overcome so that they reached a higher level of performance and accuracy.

A multi-manipulator method is addressed to realize a collaborative method using a global multi-manipulator space in terms of improving the sorting capability of robots. Another research study was carried out in order to identify the gangue’s grip point and recognize it before it reached a sorting area. Due to the short sorting time and high quality, the focus was on a benefit function of the system. From the point of view of collaboration, a Hungarian algorithm was adopted and improved to receive the results of prioritized allocation, and the solution obtained was ordered by cooperative prioritization in order of priority, resulting in an optimal process and efficient collaboration between handlers [124].

Table 5 compares fully autonomous manipulators by highlighting the success rate, the mechanism and grip adopted by each robot, and if they benefit (“🗸”) from an intervention interface.

**Figure 15:**
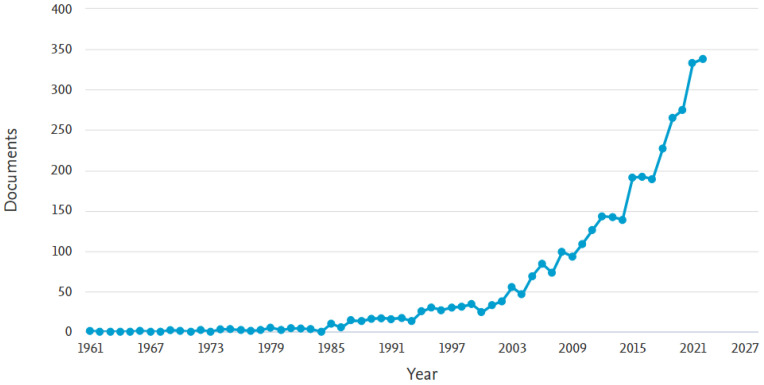
The trend in the volume of scientific literature pertaining to autonomous manipulators over time.

An analysis of the database (Figure 15) made available by Scopus between 1969 and 2022 shows the beginning of this field in 1969. A second important step when there was a substantial increase in the level of interest occured in 1987, and a high level of interest was maintained until the end of 2000. From 2000, the level of interest increased exponentially and remained the same until the end of 2022.

**Figure 16:**
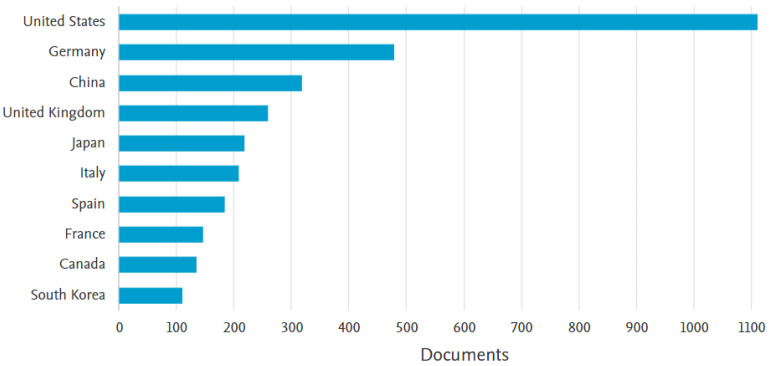
Country classification of the level of scientific interest in autonomous manipulators.

Furthermore, a graph based on data provided by Scopus shows that the United States is the market leader, so far showing the most interest in this scientific topic. Second in the ranking by a substantial gap is Germany, after which the other eight territories visible in Figure 16 show a gradual decrease in interest.

## 6. Research Challenges

In [125], attempts were made to operate autonomous robots in certain environments dangerous for users, such as those with low oxygen, explosion hazards, extremely high pressures, or even temperatures unfavorable to human life, all of which are classified as harsh environments for humans. These harsh environments can also be considered as challenging for individuals in terms of environmental unfamiliarity, dynamics, disorder, or very limited visibility.

A favorable example to explain this area is their exploitation in space, which falls squarely into the category of a harsh environment, being located at a significant distance from civilization and being largely unknown and unexplored. There is also a disadvantage in terms of transmitting information on the location of the robot, leading to significant delays due to distance. In terms of navigation, there are other issues, such as unstructured surfaces, rocks, sandy terrains, and microgravity, which hamper the system’s locomotion. Another significant problem in this area is when certain malfunctions occur within the robot, which would generate too high a cost to solve these problems. Moreover, the chances of the robot being damaged are increased due to the high probability of encountering certain unpredictable situations during its operation.

There are also a lot of activities that are carried out on the planet in unfavorable, harsh environments. Lately, the oil industry has increasingly adopted autonomous robotic systems to protect people from toxic, unsafe environments. A plausible example of such an activity is the checking of whether inland areas are filled with oil as well as the submerged outer areas of off-shore platforms, which are subject to high-risk marine conditions.

Similar to space applications are RAS (Rackspace Application Services) search-and-rescue applications, as they explore environments that are difficult or sometimes impossible for rescuers to find survivors. The situations in which these robots operate are different from action to action and depend on the environment in which they operate, which makes RAS technologies sufficiently adaptable regardless of the environment in which they operate. By adopting autonomous systems in these robots, they have managed to be more reliable, and in some cases, they are able to avoid various inconveniences, even when the user cannot access parameters due to distance or the failure of certain essential components.

An important challenge in this area is the risk of using multiple robots so that they are able to operate in teams or with other people, requiring manipulation with a large number of degrees of freedom. Additionally, in order to reach human-like activity, the robots need to improve their planning algorithms as well as properly represent their planning.

In [126], a study was conducted on the technological trends experimented with and validated so far from the point of view of autonomous cars, but also the impediments and problems that have prevented their faster evolution into a higher level of autonomy. The maps used by autonomous cars are more complex than the classic GPS-type maps, containing much more information, such as traffic signs, lane width, and the height of the road. For processing this information, the car needs a significantly large processing memory. It should be kept in mind that every kilometer traveled by the vehicle is processed and recorded in such a way that covers all territories, and recording these data requires an extremely high capacity; some attempts have already been made by obtaining data from sensors, such as 3D LIDAR, odometry, and GPS, using lower costs, but at present, mapping is still a challenge. A large amount of data needs to be retrieved and analyzed in real time from sensors, which should improve safety and driving comfort, but so far there is much left to be desired.

From the point of view of integrating autonomous robots in agriculture, there are still a number of approaches that can be implemented to improve production, and these improvements must be made to increase food quality without harming the environment. Studies in this area show that an increase in technology and new environmental trends would lead to more resilient agriculture. In terms of production, mechanized and computerized systems exist but there is a lack of digitalization and intelligence. Large machines such as tractors form a monopoly while small systems, such as robots and drones, benefiting from artificial intelligence would allow data to be taken to optimize production [127].

In [128], Chen deals with a topic that is becoming increasingly popular: the use of autonomous delivery robots (ADRs). His experiment focuses on an adaptive heuristic algorithm to which improvements are made in order to identify large neighborhoods willing to deliver up to 100 customers. A major challenge arises when ADRs cannot fulfill deliveries due to customer requirements, limited payload, or limited reach. This leads to an inability to complete the order and locks this robot in a certain way on the pavement.

The area of the deployment of autonomous robots that carry out work in underwater environments is a meticulous subject that must be treated seriously, as the dangers are much more fatal and polluting. This is why underwater missions that contain multiple phases in their execution represent additional challenges. The analysis and detection of the area of operation can take several hours due to the environment being disturbed by natural phenomena, such as currents, wildlife activities, and disturbances if in a high-activity area. This can lead to a change in the environment in which the analysis was performed, and at the time of the mission, the data analyzed and stored in view of the environment may not correspond to reality [129].

In order to improve the location method used by autonomous vehicles, the technique of combining LIDAR location sensors is used, especially for 3D localization. This sensor is one that offers excellent accuracy, but there has been an economic problem in its implementation. LIDAR is a sensor composed of several particle filters that use location and address mapping to get the most accurate results. However, due to its high cost, this method is not a very reliable one; therefore, other technologies may be used in the future [130].

Moving in hard-to-reach environments, such as certain underground caves or underwater, are topics of interest for which specialized robots have been developed. Because the environment in which they operate is difficult and imprecise, there is a need for localization and mapping technology that can provide the most accurate location details possible. Therefore, technologies have been developed to introduce SLAM modules to facilitate the localization environment by assigning metric spacing. However, the problem encountered in the development of this method was the non-application of noise resonance, which could only be achieved up to a level of 25% [131].

Map-based localization using a multi-camera system is increasingly being used by robots to operate autonomously in spaces with high human uncertainty. That is why obtaining multiple photographs based on the deployed camera system and combining them to obtain detailed mapping is a very complex method of improving accuracy. The segmentation of obtained images was performed in [132] to achieve a precise outline of the space in which a robot operates. However, even if the system works, it runs into problems if the environment is too bright or the workload is more dynamic.

In [133], six problems are highlighted that are expected to be solved when considering the development of autonomous robots:

1. The development of methods to ensure safe operation in crowded and complex environments while simultaneously modeling robot interaction with other robots’ interactions;
2. New autonomous learning solutions need to be considered in terms of decision making, and subsequently evaluated and implemented;
3. There is a need for development in terms of fleet management, the quality of services, and online performance;
4. The mode of operation in adverse weather conditions needs to be developed;
5. There is a need for the verification of methods for safety assessments;
6. It is necessary that perception and planning are closely linked in terms of the direct propagation of uncertainty.

The main challenges when considering autonomous assistive robots arise when we want to achieve an algorithm that allows a robot to behave in an easily achievable way when interacting with a new unknown environment [11,31]. In some cases, some tests have been successful in places unknown to these robots, but they were not fully in control of the system from a safety point of view [13,33,35,37,44].

A challenge that has arisen in several fields concerning the activity of autonomous robots is the development of specific algorithms to improve the safety and efficiency level in their perception of a route without colliding with certain fixed or mobile obstacles. This has been experimentally tested in activities in hospitals [10,85,86,89], in industrial spaces [36,69,70,88], and when road driving [53,55,57,58,59,60]. Another raised issue is based on the scenario where the results are hard to obtain due to the deficient collaboration between autonomous robots when they are manipulating certain objects. This is related to the data transmittion or data reception process [24,61,66,78,79,80,81,90].

From the point of view of improvements in this field of autonomous robots, there are a number of things that can be taken into account in order to achieve more comprehensive results. For all the types of robots discussed so far, it would be of added benefit to adopt or improve a neural network to learn the necessary tasks to be performed and to develop new autonomous skills in terms of the successful completion of steps and to develop control mobility in terms of safety and adaptability to the environment in which they operate. Other important aspects that should be integrated into the systems of these robots are the transmission, processing, and manipulation of data, adopting a more secure approach in terms of the data it captures, either from the user or from private environments.

Table 6 shows a comparison of all the publications discussed by highlighting the year of publication, the software/algorithm they adopted in performing the tasks, the sensors or components that receive signals from the external environment, and the type of technique used in the research.

## 7. Conclusions

In order to add to this scientific field, it is necessary to identify all the existing opportunities to date and exploit them both simultaneously and separately in order to be able to see what is the most current state of research in this area. It is observed from the data provided by Scopus that the level of interest in autonomous systems has been continuously increasing from one year to the next, covering several fields of activity in terms of human assistance. In order to bring added benefit, improvements to existing systems are being considered, such as the collaboration of assistive robots, the efficient and uninterrupted storage and processing of information, and their adoption and efficient decision-making in critical moments not yet encountered in the course of business.

## Figures and Tables

**Table 1:** Comparing the implementation of assistive robot systems.

Refs	Task	Field	Speed	Camera
[16]	Elderly care	Assistance	Real Time	CCD color
[19]	Augmented walker	Assistance	N/A	N/A
[24]	Interaction with a group of users	Assistance	Real Time	2D Axis M1031-W
[25]	Integration with a group of people	Assistance	Real Time	2D laser/RGB-D
[28]	Evaluation of human–robot interaction	Assistance, Rehabilitation	N/A	N/A
[31]	Autonomous air hockey game	Assistance	Real Time	N/A
[32]	Workplace inspection	Assistance	500 Hz	Sony PlayStation Eye
[33]	Semantic assisted trajectory planning	Assistance	Real Time	2D laser/RGB-D
[34]	Health monitoring, complementary care and social support	Assistance, Rehabilitation	Real Time	Thermal/RGB-D
[35]	Detection, tracking people in their environment	Assistance, Rehabilitation	100 Hz/7.5 Hz	2D laser/RGB-D
[36]	Educational and commercial purposes	Assistance	N/A	2D laser
[37]	Social navigation	Assistance	4Hz	2D laser/RGB-D
[38]	Therapy system	Rehabilitation	N/A	RGB-D
[40]	(3D) vision-based SLAM	Assistance	N/A	RGB-D
[41]	Robot commercial controlled with joystick	Assistance	Real Time	RGB-D
[42]	Recovering posture	Assistance, Rehabilitation	N/A	RGB-D
[43]	Interactive games with people	Assistance	Real time	N/A
[44]	Recognize gestures	Assistance	Real Time	RGB
[45]	Face detection	Assistance	10 fps	N/A
[46]	Home care	Assistance, Rehabilitation	N/A	RGB-D
[47]	Recognition of activities	Assistance, Rehabilitation	Real Time	RGB-D

**Table 2:** Autonomous cars’ characteristics.

Changes	Extended Objectives
Energy	Low-cost renewable energy
Emissions	No environmental impact at the tailpipe
Safety	Accident-free vehicles
Congestion	Congestion-free route. Easier parking.
Affordability	Vehicles suitable for any type of luggage or purpose

**Table 3:** Comparison of autonomous vehicle systems.

Refs	Radar	Lidar	Speed	Probabilities	Camera
[55]	🗸	🗸	real-time	N/A	🗸
[56]	🗸	🗸	real-time	70%	🗸
[61]	X	🗸	real-time	96–100%	🗸
[58]	🗸	X	real-time	93.24%	🗸
[63]	🗸	X	N/A	98%	🗸
[64]	🗸	X	real-time	N/A	🗸
[66]	X	X	real-time	95%	🗸
[67]	🗸	X	real-time	80.8%	🗸
[69]	🗸	🗸	real-time	N/A	🗸
[70]	🗸	🗸	N/A	N/A	🗸
[71]	🗸	🗸	real-time	N/A	🗸
[72]	🗸	🗸	real-time	N/A	🗸

**Table 4:** Comparison of autonomous transport systems.

Refs	Range	Environment	Speed	Camera
[84]	19.2 Km	Industrial	5 km/h	🗸
[85]	N/A	Warehouse	5 km/h	N/A
[86]	N/A	Industrial	1.2 m/s	N/A
[88]	N/A	Industrial	40 m/min	🗸
[89]	12 day	Industrial	45	X
[91]	95.0–137.9 cm	Industrial/home	N/A	🗸
[95]	7 h	Hospital	1.0 m/s	🗸
[96]	N/A	Hotel	N/A	🗸
[97]	1 h	Office	1.0 m/s	🗸
[98]	N/A	Industrial	N/A	🗸

**Table 5:** Comparison of autonomous manipulation systems.

Refs	Success Rate	Interface	Mechanism	Gripper
[108]	100%	N/A		
[109]	100%	🗸	A two-finger parallel	Push-to-grasp
[111]	90.5%	N/A	Cylindrical fingers, circular flat	Push-to-grasp
[112]	100%	N/A	Fingers	Push-to-grasp
[113]	100%	N/A	Afm probe	Adhesion
[114]	N/A	🗸	Two-finger	Push-to-grasp
[115]	80%	N/A	Fingers	Push-to-grasp
[116]	100%	N/A	Fingers	Push-to-grasp
[117]	N/A	🗸	EndoWrist	Push-to-grasp
[118]	N/A	🗸	Fingers	Push-to-grasp
[119]	100%	🗸	Fingers	Push-to-grasp
[120]	100%	X	N/A	Push
[121]	N/A	N/A	Fingers	Push-to-grasp
[122]	2015	X	Fingers	Push-to-grasp

**Table 6:** Comparison of all four chapters.

Refs	Year	Software/Algorithm	Sensors/Adopted	Technique
[16]	2005	Tele-presence interface, speech interface, face finding and tracking, navigation	Sonar, touch, position, camera,	Simulation
[19]	2003	Mapping and motion	For position, for components	Simulation
[24]	2014	ROS	ASUS Xtion PRO LIVE, 2D Axis M1031-W, 2D Axis M1031-W	Measurement
[25]	2022	Detecting groups, estimating F-formations,	Laser, camera	Simulation
[28]	2011	Meta-analytic	N/A	Measurement
[31]	2019	localPathCorrection/ localPathCorrection	N/A	Measurement
[33]	2019	RRT	Camera	Measurement
[34]	2020	Innovative perception and interaction capabilities	Monitoring, 2D laser scanners	Measurement/Simulation
[35]	2015	Munkres/ Leg Tracker	Laser, noise	Measurement/Simulation
[36]	2020	ROS	Ultrasonic, s RPLiDAR, IMU, encoder, camera	Simulation
[37]	2021	GMapping/ SLAM	Hokuyo UTM-30LX Laser Rangefinder, RGB-D, noises, camera	Simulation
[38]	2016	SVM/ Random Forest/ AdaBoost	Camera	Measurement
[40]	2014	RANSAC	Laser range finders, sonars, cameras, radars, inertial	Simulation
[41]	2019	Processing-based position/pose	RGB-D, Intel RealSense D435, infra-red, camera	Simulation
[42]	2016	DCSF	Kinect, RGB-D Cameras	Simulation
[43]	2010	CAMSHIFT	LEDs, camera	Measure/Simulation
[44]	2016	DTW	Kinect, vision, camera	Measure
[45]	2018	Tracking, vision algorithm	Camera	Measure
[46]	2018	Pose/skeleton recognition	Laser, accelerometer, camera, microphones, infrared	Simulation
[47]	2020	Particle filter, clustering	RGB-D camera	Simulation
[48]	2018	Safety reasoning and casualty minimization	radar, lidar, cameras, gps, v2x	Simulation
[61]	2015	Improving the efficiency and quality of sensor data fusion	lidar, radar and camera	Measure/Simulation
[62]	2007	Detection/recognition of the sign, tracking	camera	Simulation
[63]	2016	Trained a convolutional neural network (CNN) to map raw pixels	Cameras	Simulation
[64]	2009	LfD	-	Simulation
[65]	2019	Horizon, heuristic	-	Measure/Simulation
[66]	2010	Visual odometry	Camera, GPS	Measure/Simulation
[67]	2018	FollowerStopper	OBD-II, Camera	Simulation
[68]	2020	Vehicle density iteratively, flow-density plots	N/A	Simulation
[70]	2017	3D perception, state estimation and data fusion, 3D perception, state estimation and data fusion	N/A	Measure/Simulation
[71]	2019	Mapping, mapping, artificial intelligence, planning	Tactile, Tactile, Encoders, Encoders, Ultrasonic, Sonar, Accelerometers, Gyroscopes	Measure/Simulation
[72]	2018	Planning, Dijkstra, Bellman–Ford, Floyd, control	Control, radar, radar	Simulation
[84]	2021	-		Simulation
[86]	2020	Planning and motion coordination	laser scanner and odometry sensors	Simulation
[88]	2008	Navigational	Proprioceptive and exteroceptive	Simulation
[90]	2000	Spreading activation	CMs, FDs	Simulation
[91]	2006	Evolutionary	Exteroceptive, proprioceptive	Simulation
[92]	1993	Decentralized	Force	Measure
[83]	2002	Control	Trajectory	Simulation
[94]	2013	-	Ultrasonic sensor, RFID, QR-code and camera sensor	Simulation
[95]	2012	-	LRF, Camera	Simulation
[96]	2010	Far approach, Near-approach, Stair alignment, Stair traversal	Inertial, Camera	Simulation
[97]	2010	Path planning, virtual potential field	LRF, ultrasonic, stereo vision	Simulation
[88]	2009	Fitting, detection	N/A	N/A
[109]	2017	Perception, Planner	Actuators, RBG-Leds, pose, accelerometers	Simulation
[110]	2012	Autonomous manipulation	Actuators, kinesthetic	Simulation
[111]	2014	Newton–Euler	Barrett, Barrett WAM	Measure/Simulation
[112]	2014	Segmentation	RGB-D camera, Kinect, 3-D noise, orce/torque	Measure
[113]	2014	RGB-D	Facet detection, segmentation	Simulation
[115]	2019	Control, visual tracking, Lucas–Kanad	camera	Simulation
[116]	2011	Planning, visual tracking	cameras	Simulation
[117]	2009	Controls	Depth, f orce/torque, orce/torque	Simulation/Measure
[118]	2015	Panel localisation, vision	navigatio, video camera, sound velocit	Simulation/Measure
[119]	2003	Detection, grasping	force and visual	Simulation
[103]	2015	ICP, perception	Coordinates, Calibration	Simulation/Measure
